# A novel technique of laparoscopic reduction of incarcerated internal supravesical hernia via peritoneal incision: A case report

**DOI:** 10.1016/j.ijscr.2020.09.074

**Published:** 2020-09-15

**Authors:** Yugo Matsui, Teppei Murakami, Kenta Horita, Satoshi Ishida, Shotaro Matsuda, Aoi Tayama, Ryutaro Sakata

**Affiliations:** Department of Surgery, Kobe City Medical Center West Hospital, 2-4 Ichibancho, Nagata-ku, Kobe, 653-0013, Japan

**Keywords:** Internal supravesical hernia, Laparoscopy, Laparoscopic reduction, Small bowel obstruction, Peritoneal incision technique

## Abstract

**Introduction:**

Laparoscopic reduction techniques for internal supravesical hernia have not been discussed much in literature.

**Case presentation:**

A 90-year old woman was admitted for symptoms of intestinal obstruction. She was diagnosed with small bowel strangulation by CT scan and laparoscopy was performed. Laparoscopy revealed a mass medial to the medial inguinal fold with tightly incarcerated small bowel. The bowel could not be reduced by traction or external compression, and required incision of the hernia ring. The tight incarceration posed a risk of bowel injury and so we performed peritoneal incision in a similar manner to the TAPP approach for loosening and precise incision of the hernia ring. The bowel was successfully reduced and the hernia was repaired by partial sac resection.

**Discussion:**

Surgical methods as well as reduction technique were reviewed from previous literature. Procedures with open laparotomy, laparoscopy and anterior approach have been described, but details of reduction were not seen in many of these reports. Various methods have been described for bowel reduction in other hernias, but none involving peritoneal incision. This is the first report describing bowel reduction via the peritoneal incision technique.

**Conclusion:**

Internal supravesical hernia may pose difficulty in bowel reduction, but the peritoneal incision technique allows safe incision of the hernia ring under laparoscopic situations.

## Highlights

• There is no golden standard for surgical procedure for internal supravesical hernia.
• There are previous reports of surgery by open laparotomy, laparoscopy and anterior approach.
• Various methods for bowel reduction have been described for other hernias, but none by peritoneal incision.
• The incarcerated small bowel was safely reduced by peritoneal incision in our case.

## 1 Introduction

Internal supravesical hernia is a rare condition with no golden standard for surgical procedures. Cases of surgical repair by open laparotomy, laparoscopy, and anterior approach have been previously reported, but not many include details of how the incarcerated bowel was reduced. We hereby report a case of internal supravesical hernia with tight incarceration of small bowel successfully reduced via laparoscopy by the peritoneal incision technique. This work has been reported in line with the SCARE criteria [1].

## 2 Case presentation

**Fig. 1:**
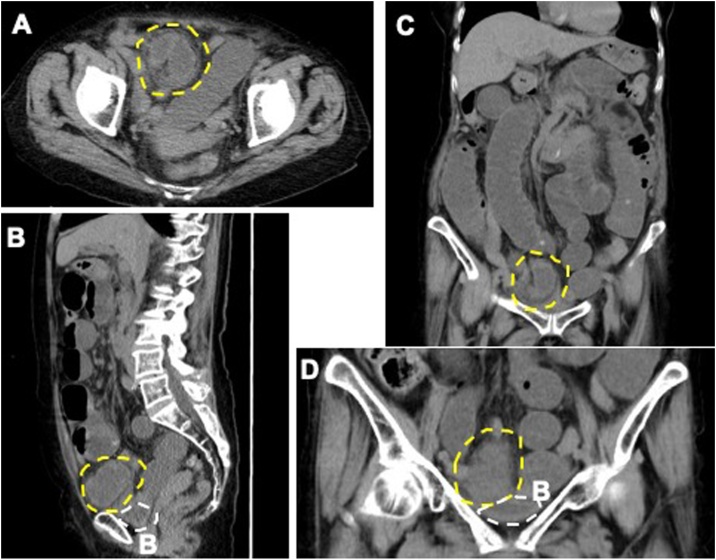
CT scan revealing a sac-like mass involving a dilated bowel loop (yellow dotted lines) descending anteriorly and laterally with respect to the bladder (B/white dotted line). Axial (A), sagittal (B) and coronal (C,D) sections.

A 90-year-old woman with no history of abdominal surgery presented with vomiting and lower abdominal pain. On examination, she was afebrile with normal vital signs. Her abdomen was significantly distended with tenderness near her umbilicus. Laboratory tests revealed an elevation in inflammatory markers (white blood cell count of 8300/μL and a C-reactive protein level of 6.49 mg/dL), but a normal lactate level. Contrast computed tomography showed a small bowel obstruction with a transitional zone in the right lower abdomen. A right indirect herniation could be seen, but there was no bowel involvement and thus was not considered to be the cause of obstruction (Fig. 1). She was hospitalized for small bowel obstruction of unclear origin. Although inflammatory markers improved the next day, symptoms worsened, and urgent laparoscopy was performed on the same day for suspected bowel strangulation.

**Fig. 2:**
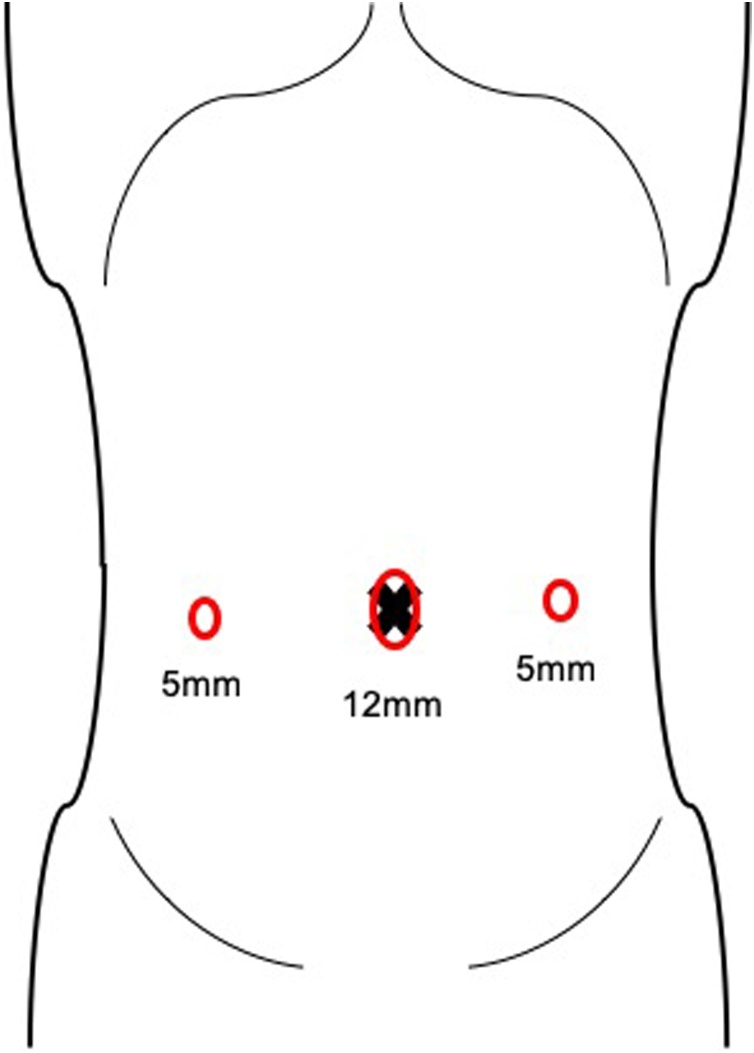
Schematic illustration of port placement.

**Fig. 3:**
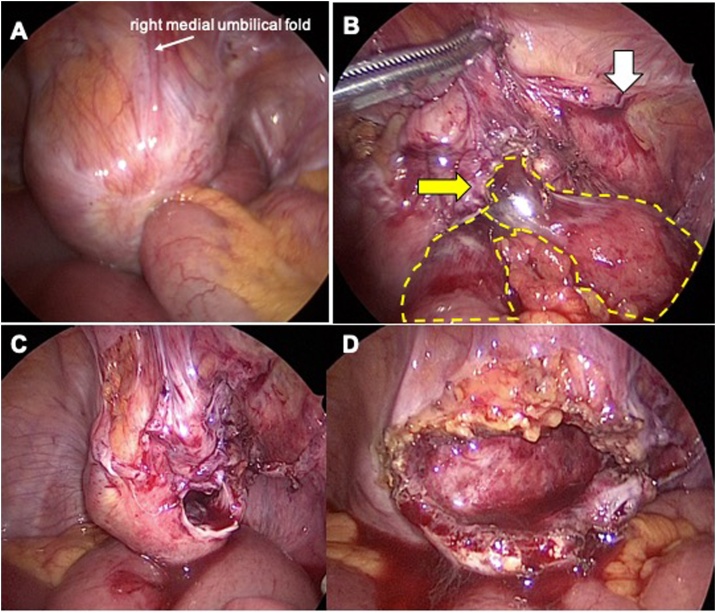
Intraoperative laparoscopic images showing incarcerated small bowel in a hernia sac located medial to the right medial umbilical fold (A). Incision of the peritoneal membrane began lateral to the sac (white arrow) and extended to the orifice, which allowed loosening and a safer incision of the orifice (yellow arrow). Incarcerated bowel shown by yellow dotted lines (B). View after reduction (C). The sac was partially resected (D).

A laparoscopy with a three-trocars technique (12 mm at navel and two 5 mm trocars at the bilateral abdominal flank) was performed (Fig. 2). Upon inspection, a large bulging mass was seen medially to the right medial umbilical fold with incarceration of small bowel (Fig. 3A). This attachment was found to be the obstruction site, and an incarcerated internal supravesical hernia was suspected. The bowel was tightly incarcerated and could not be reduced via traction or exterior compression. Incision of the hernia ring was necessary for reduction, but there was no space between the bowel and the orifice wall. We decided to dissect the peritoneum in a manner similar to the transabdominal pre-peritoneal (TAPP) approach for inguinal hernia repair, in order to distinguish the peritoneum from any content inside the sac. We began peritoneal incision near the medial inguinal fossa and extended it towards the hernia ring. The peritoneum was mobilized prior to each incision. With this wide incision, the orifice loosened allowing space for a precise incision of the orifice, and the bowel was reduced without injury (Fig. 3B). This revealed a hernia ring of approximately 2 cm × 2 cm (Fig. 3C). There was a concern of abscess formation since the sac contained inviable bowel, and so we chose to partially resect the sac to prevent fluid accumulation (Fig. 3D). The naval trocar site was extended roughly 3 cm and the gangrenous bowel was resected with a stapling device. The proximal and distal bowel was viable, and a functional end-to-end anastomosis was performed. Operation time was 143 minutes with trivial bleeding. The patient was discharged on post-operative day 18, since a worsened rectal prolapse required surgery during her stay. No recurrence was seen 12 months later.

## 3 Discussion

Internal supravesical hernia is a rare condition, first reported by Ring in 1814 [2]. It occurs in the triangular space surrounded laterally by the medial umbilical ligament, medially by the median umbilical ligament (urachas), and inferiorly by the transverse vesical fold. The supravesical hernia is either external or internal depending on the direction of sac extension. External supravesical hernia protrudes into the inguinal canal mimicking a direct inguinal hernia, whereas an internal supravesical hernia remains inside the abdomen [3,4].

The rarity of this condition can be seen from the fact that there are only 41 results in a Pubmed search with the MeSH term “internal supravesical hernia”. Of these results, 16 cases were found with operative details (Table 1) [[5], [6], [7], [8], [9], [10], [11], [12], [13], [14], [15], [16], [17], [18]]. The median age of these reports is 62 years old, with 13 out of 17 cases occurring in males, inferring that males in their 60’s is an epidemiological risk. Patients typically present with symptoms of intestinal obstruction, but there are also reports on patients with bladder symptoms [19]. Pre-operative diagnosis is often difficult with only one case having an accurate diagnosis in Table 1 (emphasized with bold text). Typical CT findings of a dilated bowel loop trapped in the supravesical fossa compressing the bladder have been described in hope of improving accuracy in pre-operative diagnosis, but the lack of awareness to this rare condition is a big obstacle in achieving this goal [20,21]. Patients are usually diagnosed with small bowel obstruction/strangulation (8/17 cases, 47%) or internal hernia (3/17 cases, 17.6%), and undergo urgent surgery. Hence, the low accuracy in pre-operative diagnosis does not actually have a great impact on mortality. However, it frequently involves necrosis of the incarcerated bowel, with 6 out of 17 (35%) in our literature review, and so awareness of the importance of urgent surgical attention is essential.

**Table 1:** Literature review.

Author	Age/Sex	Presenting complaint	Preoperative diagnosis	Method of operation	Closure of sac	Sac resection	Intestinal resection	Reduction technique
Koksoy F [5]	78/Male	abdominal pain, vomiting	small bowel obstruction	laparotomy	yes	no	no	unknown
Yamaguchi R [6]	74/Male	abdominal pain, vomiting	small bowel obstruction	laparotomy	yes	yes	no	unknown
Gorgun E [7]	unknown	unknown	small bowel invagination	laparoscopy	yes	no	no	unknown
Mehran A [8]	52/Male	abdominal pain, vomiting	small bowel obstruction	laparoscopy	yes	no	yes (necrosis)	unknown
Sozen I [9]	43/Male	abdominal pain, vomiting	small bowel obstruction	laparotomy	no	yes	no	unknown
Saravanan B [10]	62/Male	abdominal pain, vomiting	small bowel obstruction	laparotomy	yes	no	yes (necrosis)	unknown
Jan YT [11]	75/Male	abdominal pain, vomiting	internal hernia	laparotomy	yes	no	no	unknown
Cisse M [12]	60/Male	abdominal pain, vomiting	small bowel intussusception	laparotomy	yes	no	no	cautious traction
Schwarz L [13]	76/Male	abdominal pain, vomiting	small bowel intussusception	laparotomy	yes	yes	yes (necrosis)	unknown
Bouassida M [14]	58/Female	abdominal pain, vomiting	internal supravesical hernia	laparotomy	yes	no	yes (necrosis)	unknown
	36/Male	abdominal pain, vomiting	small bowel obstruction	laparotomy	yes	yes	no	slight traction
Sanchez-Fuentes PA [15]	74/Male	abdominal pain, distention	small bowel obstruction	laparotomy	yes	no	no	unknown
Khalid S [16]	62/Male	abdominal distention, vomiting	small bowel obstruction	laparotomy	yes	no	no	gentle traction
Morimoto M [17]	75/Male	abdominal pain, nausea	strangulated inguinal hernia	anterior approach	yes	yes	yes (necrosis)	sac incision
Marco C [18]	48/Male	abdominal pain, vomiting	internal hernia	laparoscopy	yes	no	no	gentle traction
	65/Male	abdominal pain, vomiting	internal hernia	laparoscopy	yes	no	no	unknown
Our case	90/Female	abdominal pain, vomiting	small bowel strangulation	laparoscopy	no	yes	yes (necrosis)	peritoneal incision of sac

Due to the rarity of the condition, there is no golden standard for surgical procedure in internal supravesical hernia, but trends can be seen from our literature review. First of all, the hernia orifice was closed with sutures in 15 out of 17 cases (88%), which is thought to be important in preventing recurrence. We did not close the orifice, partly due to our ignorance of the condition and its trending surgical procedure, but also due to concerns of abscess formation. However, post-operational abscess formation has not been documented for internal supravesical hernia, so perhaps it should not have been worried for. Instead of closing the orifice, our strategy for preventing recurrence of bowel obstruction was to partially resect the sac. Sac resection was only done in 2 out of the 17 cases, and is thought to be unnecessary. In fact, Marco et al. discuss that sac dissection poses the risk of bladder damage [18]. In our case, the sac had significant protrusion into the abdomen allowing for dissection without injury to the bladder. Recurrence has not occurred 12 months after surgery, which may be an acceptable outcome.

Reduction of the incarcerated bowel is an essential step in surgical treatment of incarcerated hernia, whether it be internal supravesical or inguinal. Details of reduction were described in only a few cases in Table 1 with traction being the only technique described. In our case, this seemed impossible without bowel injury. External compression was ineffective since pressure could not be sufficiently applied to a herniation not protruding through the abdominal wall. Other techniques have been described for inguinal, obturator and femoral hernia, such as the water pressure method and releasing incision technique [[22], [23], [24]], but methods dissecting the peritoneum have not been reported so far in Pubmed (MeSH term: laparoscopy, reduction, hernia, peritoneal, incision). Our case is the first to apply this method, succeeding in bowel reduction without injury. Peritoneal incision is done in a fashion similar to the TAPP approach of inguinal hernia repair allows precise mobilization and incision of the peritoneum. We believe that incision can begin anywhere as long as the operator can be sure that only the peritoneum is being mobilized. We began at the medial inguinal fossa, since it seemed to be closest point to the orifice without involvement of the hernia sac. This technique is thought to be particularly useful in reduction of internal hernias where external compression of the bowel is difficult or impossible. However, it must be noted that this method may be contraindicated in cases of incarcerated inguinal, femoral or obturator hernia involving bowel resection with elective mesh repair, since exposure of the preperitoneal space to the gangrenous bowel could lead to mesh infection.

## 4 Conclusion

The peritoneal incision technique allows laparoscopic reduction of tightly incarcerated supravesical hernia.

## Declaration of Competing Interest

None.

## Funding

None.

## Ethical approval

This case is not a research study.

## Consent

Written informed consent was obtained from the patient for publication of this case report and accompanying images. A copy of the written consent is available for review by the Editor-in-Chief of this journal on request.

## Author contribution

Yugo Matsui: Responsible for literature review, writing and manuscript preparation. Operator of the surgery.

Teppei Murakami: Responsible for manuscript preparation.

Kenta Horita: Assisted in the laparoscopic surgery.

Shotaro Matsuda, Aoi Tayama: Responsible for manuscript review

## Registration of research studies

This is not a research study.

## Guarantor

Yugo Matsui.

## Provenance and peer review

Not commissioned, externally peer-reviewed.
